# Prone during pandemic: development and implementation of a quality-based protocol for proning severe COVID-19 hypoxic lung failure patients in situationally or historically low resource hospitals

**DOI:** 10.1186/s12890-021-01401-0

**Published:** 2021-01-12

**Authors:** Alfredo J. Astua, Eli K. Michaels, Andrew J. Michaels

**Affiliations:** 1 Division of Pulmonary and Critical Care Medicine, NYCHHC/Elmhurst Hospital Center, New York City, USA; 2 Division of Epidemiology, University of California, Berkeley School of Public Health, Berkeley, CA USA

**Keywords:** Acute respiratory distress syndrome (ARDS), COVID-19, SARS CoV-2, Proning, Prone positioning, High reliability organization (HRO), Crew resource management (CRM), Low resource setting, Austere environment, Low- and middle-income countries (LMICs)

## Abstract

**Background:**

Intermittent Prone Positioning (IPP) for Acute Respiratory Distress Syndrome (ARDS) decreases mortality. We present a program for IPP using expedient materials for settings of significant limitations in both overwhelmed established ICUs and particularly in low- and middle-income countries (LMICs) treating ARDS due to COVID-19 caused by SARS CoV-2.

**Methods:**

The proning program evolved based on the principles of High Reliability Organizations (HROs) and Crew Resource Management (CRM). Patients with severe ARDS [PaO_2_:FiO_2_ ratio (PFr) ≤ 150 on FiO_2_ ≥ 0.6 and PEEP ≥ 5 cm H_2_O] received IPP. Patients were placed prone 16 h each day. When PFr was ≥ 200 for > 8 h supine IPP ceased. IPP used available materials without requiring additional work from the bedside team. Changes in PFr, PaCO_2_, and the SaO_2_:FiO_2_ ratio (SaFr) positionally were evaluated using t-statistics and ANOVA with Bonferroni correction (*p* < 0.017).

**Results:**

Between 14APR2020 and 09MAY2020, at the peak of deaths in New York, there were 202 IPPs in 29 patients. Patients were 58.5 ± 1.7 years of age (37, 73), 76% male and had a body mass index (BMI) of 27.8 ± 0.8 (21, 38). Pressor agents were used in 76% and 17% received dialysis. The PFr prior to IPP was 107.5 ± 5.6 and 1 h after IPP was 155.7 ± 11.2 (*p* < 0.001 compared to pre-prone). PFr after the patients were placed supine was 131.5 ± 9.1 (*p* = 0.02). Pre-prone PaCO_2_ was 60.0 ± 2.5 and the 1-h post-prone PaCO_2_ was 67.2 ± 3.1 (*p* = 0.02). Supine PaCO_2_ after IPP was 60.4 ± 3.4 (*p* = 0.90). The SaFr prior to IPP was 121.3 ± 4.2 and the SaFr 1 h after positioning was 131.5 ± 5.1 (*p* = 0.03). The post-IPP supine SaFr was 139.7 ± 5.9 (*p* < 0.001). With ANOVA and Bonferroni correction there were statistically significant changes in PFr (*p* < 0.001) and SaFr (*p* < 0.001) and no significant changes in PaCO_2_ over the four time points measured. Using regression coefficients, the SaFrs predicted by PFrs of 150 and 200 at baseline are 133.2 and 147.3, respectively.

**Conclusions:**

An IPP program for patients with COVID-19 ARDS can be instituted rapidly, safely, and effectively during an overwhelming mass casualty scenario. This approach may be equally applicable in both traditionally austere environments in LMICs and in otherwise capable centers facing situational resource limitations.

## Background

The use of prone positional therapy for severe hypoxic Acute Respiratory Distress Syndrome (ARDS) is known to decrease mortality by approximately one half [1, 2]. Although there are atypical aspects to the hypoxemic lung failure caused by COVID-19 [3, 4] (Corona Virus Disease 2019, SARS CoV – 2), these patients respond to positional therapy much like other patients with moderate-severe hypoxemic ARDS [PaO_2_:FiO_2_ ratio (PFr) < 150] [5].

There have been many barriers to the routine use of Intermittent Prone Positioning (IPP) for patients with ARDS and yet, throughout the world, medical facilities are being overwhelmed with hypoxic patients due to COVID-19 [5]. Our goal was to institute a positional therapy program during a period of extreme institutional stress and to adapt the procedure to be applicable in settings of significant limitations and austerity.

We present the evolution of a high reliability protocolized program for prone positioning using minimal materials in NYCHHC/Elmhurst Hospital in New York City during early April 2020 at the peak of the overwhelming surge of COVID-19 patients. This work was conceived and implemented with a focus on small team dynamics and performance improvement based in the principles of High Reliability Organizations [6] (HROs) and Crew Resource Management [7] (CRM). At the time of the spring COVID surge, NYCHHC/Elmhurst had no experience with positional therapy for adults with ARDS. Our goals were to demonstrate the feasibility of a proning program under these conditions, to demonstrate the expected improvement of oxygenation with IPP in COVID patients and, by building the program around CRM and HRO principles, to normalize short loop quality cycles for patient safety and team performance improvement.

These methods may be applicable in other centers that recognize the need for safe, effective positional therapy but have not developed a means of doing so. This is a proof of concept manuscript of a rapid, quality-based method for the implementation of prone positioning in a chronically or acutely resource limited and challenged environment particularly overwhelmed hospitals and in low- and middle-income countries (LMICs).

## Methods

In the second week of April 2020 this project was conceived, designed, and implemented. The development of the program evolved through a series of many short loop quality-based changes made and evaluated in real time. These norms of immediate debriefing and concurrent data collection, evaluation, and utilization for both process and clinical decision making utilizing the principles of HROs and CRM guided the program. We encouraged a flattened hierarchy and non-punitive feedback with a focus on the traits of HROs. These principles enabled the team to operationalize the primary goals with the speed and safety required under the conditions at NYCHHC/Elmhurst during early April 2020.

### Respiratory care

Due to the resource scarcity the patients were supported by several types of ventilators including the Puritan Bennet 840® (Medtronic, Minneapolis MN), the Dräger Evita V500® (Draeger Inc. Telford PA), the Maquet Servo-i® (Getinge LLC, Wayne NJ), the Respironics V60®, the Phillips EVO Trilogy (both Koninklijke Philips N.V.), and the LTV 1000® (Vyaire, Mettawa IL). Standard principles of protective ventilation strategies including the limitation of plateau airway pressures to < 30 cm H_2_O and tidal volumes of 6–8 cc/kg ideal body weight guided therapy. Moderate hypercapnia was tolerated provided the pH was > 7.20.

### Positional therapy

Consultation to the prone team was made at the discretion of the primary treatment teams. Patients were placed in the prone position for 16 h and supine for 8 h each day as described in the Proning Severe ARDS Patients (PROSEVA) trial [2]. When their PFr was ≥ 200 for > 8 h supine, their positional therapy ceased. This protocol was designed specifically for ventilated patients. Non-ventilated patients were treated under different positional care approaches and are not reported here.

Patients were enrolled in the protocol if their PaO_2_:FiO_2_ ratio (PFr) was ≤ 150 on an FiO_2_ ≥ 0.6 and a positive end expiratory pressure (PEEP) ≥ 5 cm H_2_O provided they were between 18 and 75 years of age and had not been mechanically ventilated for more than 14 days, did not have unmanaged abdominal compartment syndrome, a BMI > 35 or a pacemaker insertion within the prior 48 h. These decisions for exclusion were primarily due to the volume of patients presenting with severe hypoxia and some patients who met criteria were excluded due to a full proning team census.

The prone team was designed to provide positional therapy in support of the primary ICU teams and consists of five to seven persons: four to six to roll the patient and one to control the airway. The airway is managed by either an intensivist or a Certified Registered Nurse Anesthetist (CRNA) deployed by the military and four to six military medics conduct the movement. Two teams work 12-h shifts each. The initial training of these teams consisted of short lectures and a three-hour hands-on simulation. Subsequent medics and non-medical team members received training at the bedside. Intensivists attended each positional change to assure compliance with the process, facilitate rapid debriefings, institute quality adjustments, and manage critical physiologic changes in the patient. In a mature form, the team will consist of a respiratory therapist (RT) for the head and bedside nurses (RNs) for the roll. Nonmedical personnel can also fill these roles but will be less capable of responding to emergencies.

Current ventilator settings were maintained except for an increase to an FiO_2_ of 100% for ten minutes of preoxygenation before each positional change. The endotracheal tubes (ETTs) were secured with an Anchorfast Oral Endotracheal Tube Fastener® (Hollister, Libertyville IL) and maintained in the midline. Further details of the protocol and procedure, including checklists, are listed in Additional file 1: appendices B, C and D.

### Data and process management

Arterial blood gases were measured at four time points; one hour prior to the first movement from supine to prone (pre-prone), one hour after the patients were placed prone (post-prone), one hour prior to being returned to the supine position (prone hour 15, pre-supine) and one hour after being placed supine (post-supine). These ABGs provided the measures of PaO_2_, PaCO_2_ and FiO_2_ and enabled calculation of the PaO_2_:FiO_2_ ratio (PFr) and the SaO_2_:FiO_2_ ratio (SaFr). These parameters are presented for the first prone/supine event only.

Data were collected concurrently by prospective design to inform and evolve the quality of the project. In addition to patient related data, every maneuver was followed by a debriefing during which quality issues identified were processed, recorded and addressed in a short loop cycle. The cohort is restricted to all patients who had complete data on arterial blood gases across all four proning stages (pre-prone, post-prone, pre-supine, post-supine) (n = 29). Data are reported as number total (n) and percent (%) or mean ± standard error of the mean (SEM) and range.

### Statistical analysis

Changes in PaCO_2_, PF ratios, and SaF ratios between proning stages are evaluated using t-statistics from a simple linear mixed model and repeated measures ANOVA, which accommodate observations that are dependent within the same subject but independent between subjects. The t-statistic tests for difference in mean of each characteristic compared to baseline (pre-prone) and the F-statistic tests the null hypothesis that the measurements are all equal across all four proning stages (e.g., PFr H_0_: PFr _pre-prone_ = PFr _post-prone_ = PFr _pre-supine_ = PFr _post-supine_). All results are reported with a test-statistic, *p*-value, and indication of statistical significance with Bonferroni correction for multiple comparisons (*p* < 0.05 / 3 comparisons = *p* < 0.017). We also perform a non-parametric permutation test to ensure our findings are robust to violations of the ANOVA’s distributional assumptions. P-values derived from the non-parametric permutation were similar (available upon request), so only the results of the ANOVA are reported. Lastly, Pearson’s correlation and simple linear regression are used to examine associations of PFr and SaFr at one hour pre-prone. Data were evaluated using R® Version 1.2.1335 (R Foundation for Statistical Computing, Vienna Austria). According to the policy activities that constitute research at the NYCHHC/Elmhurst Hospital Center, this work met criteria for operational improvement activities exempt from ethics review [8].

## Results

At the time of reporting, 40 patients were referred, and 32 patients have been treated with prone positioning. The patients were treated between April 14th, 2020 and May 9th, 2020. The peak of COVID-19 related deaths in New York was the 15th of April 2020. At the time of this report there have been 202 movements to the prone position and an equal number to the supine position. Individual patients have received between 1 and 17 (6.6 ± 0.86) prone/supine events. Currently there are 12 patients receiving positional therapy each day. Patient demographics and baseline lab values are presented in Table 1.

**Table 1:** COVID-19 patient characteristics at baseline (n = 29)

Age (years)	
Mean (SEM)	58.5 (1.7)
Range	37.0, 73.0
NA	1 (3.4)
Sex (n (%))	
Female	7 (24.1)
Male	22 (75.9)
BMI	
Mean (SEM)	27.8 (0.8)
Range	21.0, 38.0
White blood cell count (10^3^/mcL)	
Mean (SEM)	17.1 (1.6)
Range	5.7, 48.4
NA	2 (6.9)
D dimers (ng/mL)	
Mean (SD)	4762.4 (1738.5)
Range	386.0 to 47,485.0
NA	2 (6.9)
Creatine (mg/dL)	
Mean (SEM)	1.5 (0.2)
Range	0.3 to 4.9
NA	1 (3.4)
Dialysis (n (%))	
No	23 (79.3)
Yes	5 (17.2)
NA	1 (3.4)
Pressor (n (%))	
No	6 (20.7%)
Yes	22 (75.9%)
NA	1 (3.4%)

The basic ventilator settings and respiratory values at the time of consultation were an FiO_2_ of 0.79 ± 0.03 (range 0.5 to 1.0), a PEEP of 10.1 ± 0.6 (range 5 to 18 cm H_2_O), an SaO_2_ of 93.3 ± 0.85% (range 78.3 to 99%) and a PaO_2_ of 84.65 ± 5.2 (range 53 to 157). Several modes of ventilation were used including Airway Pressure Release Ventilation (APRV), Assist Control Pressure Control (ACPC), Assist Control Volume Control (ACVC) and volume control. At the time of consultation for prone positional therapy the patient’s arterial blood gas analyses were pH 7.28 ± 0.02, PaCO_2_ of 59.0 ± 2.5 mmHg, PaO_2_ 84.7 ± 5.2 mmHg, HCO_3_ of 27.8 ± 1.0 mmol/L.

**Fig. 1:**
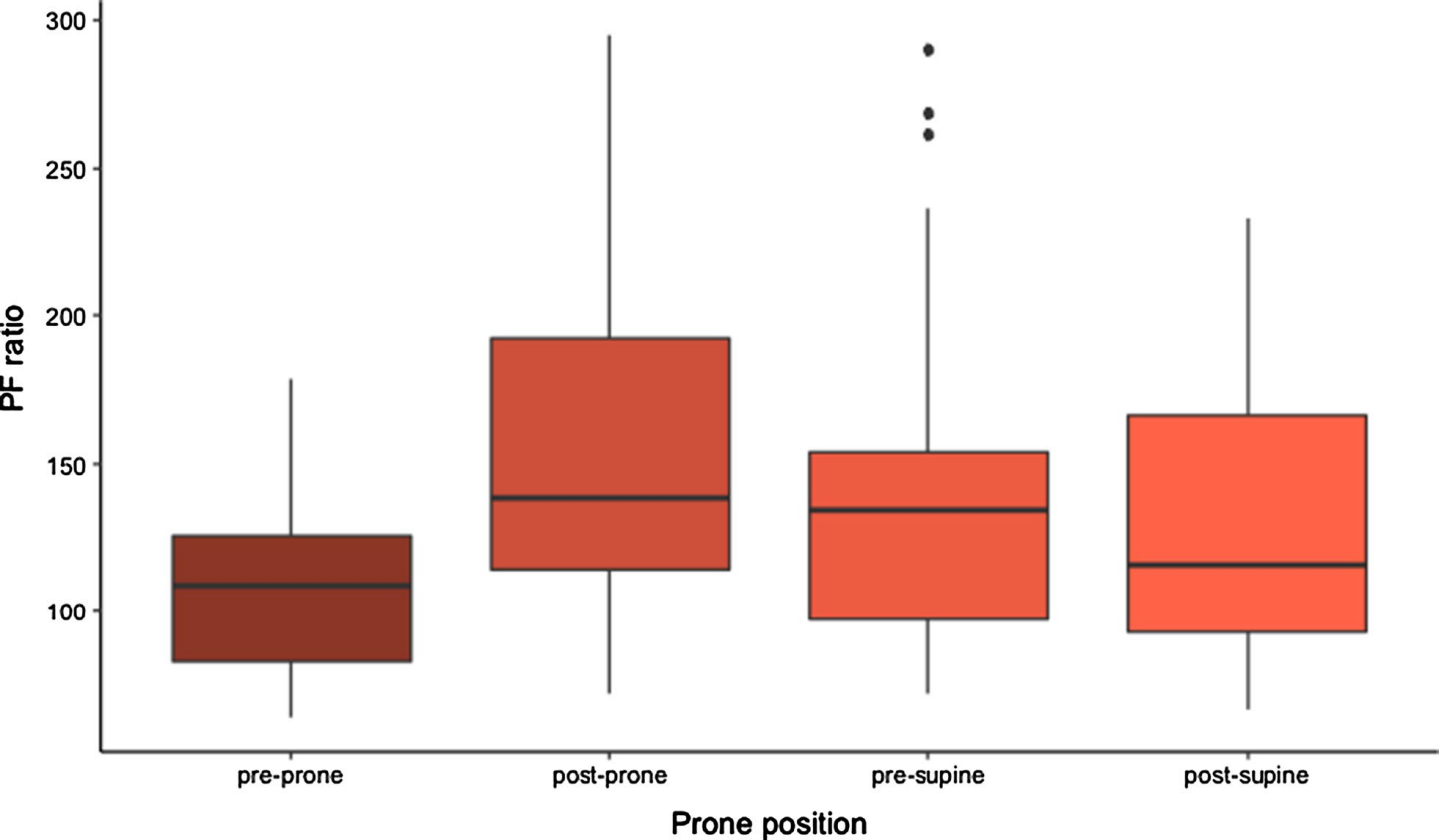
PF ratio of COVID-19 patients treated with prone positioning (n = 29)

**Fig. 2:**
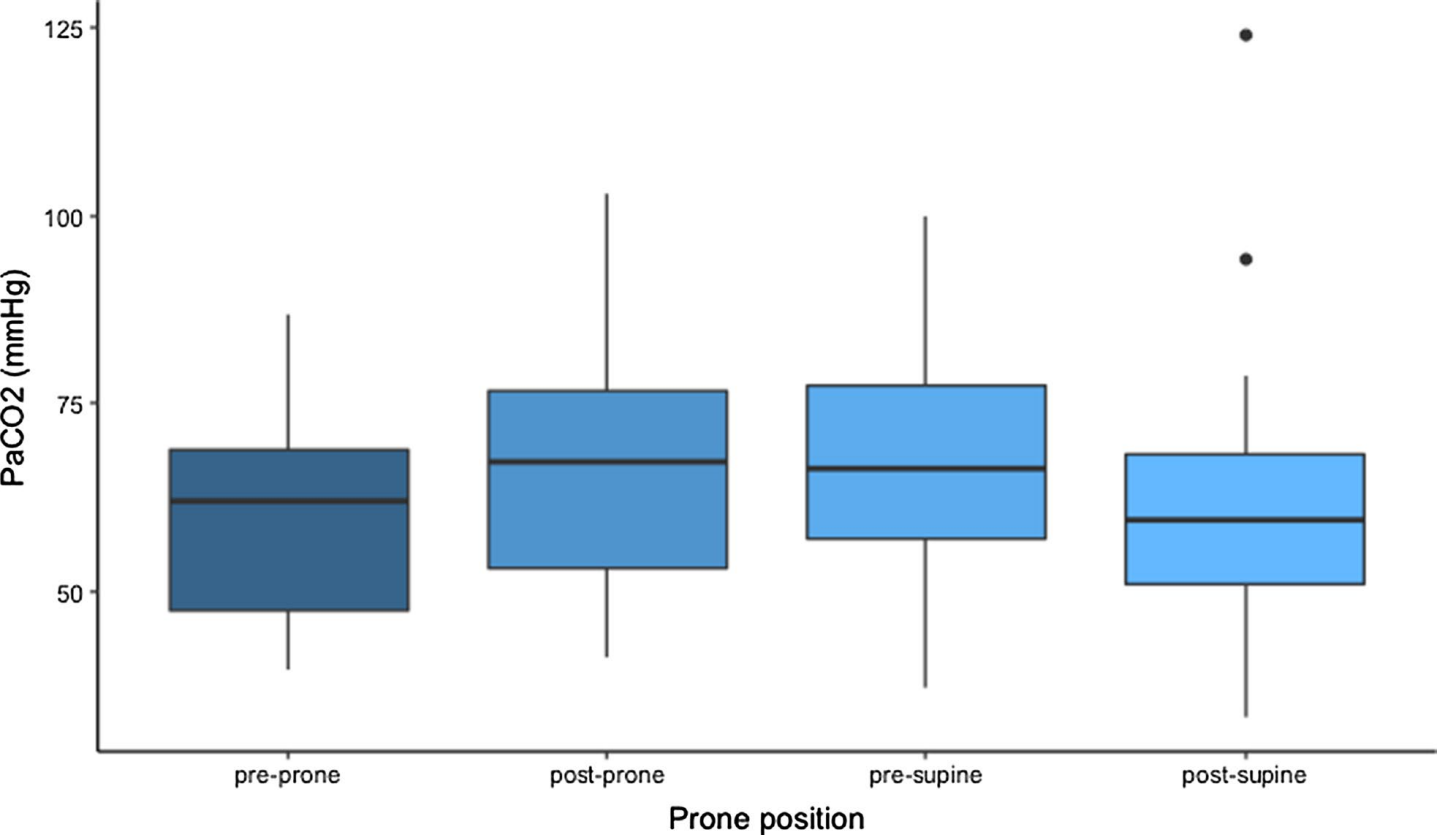
PaCO_2_ of COVID-19 patients treated with prone positioning (n = 29)

**Fig. 3:**
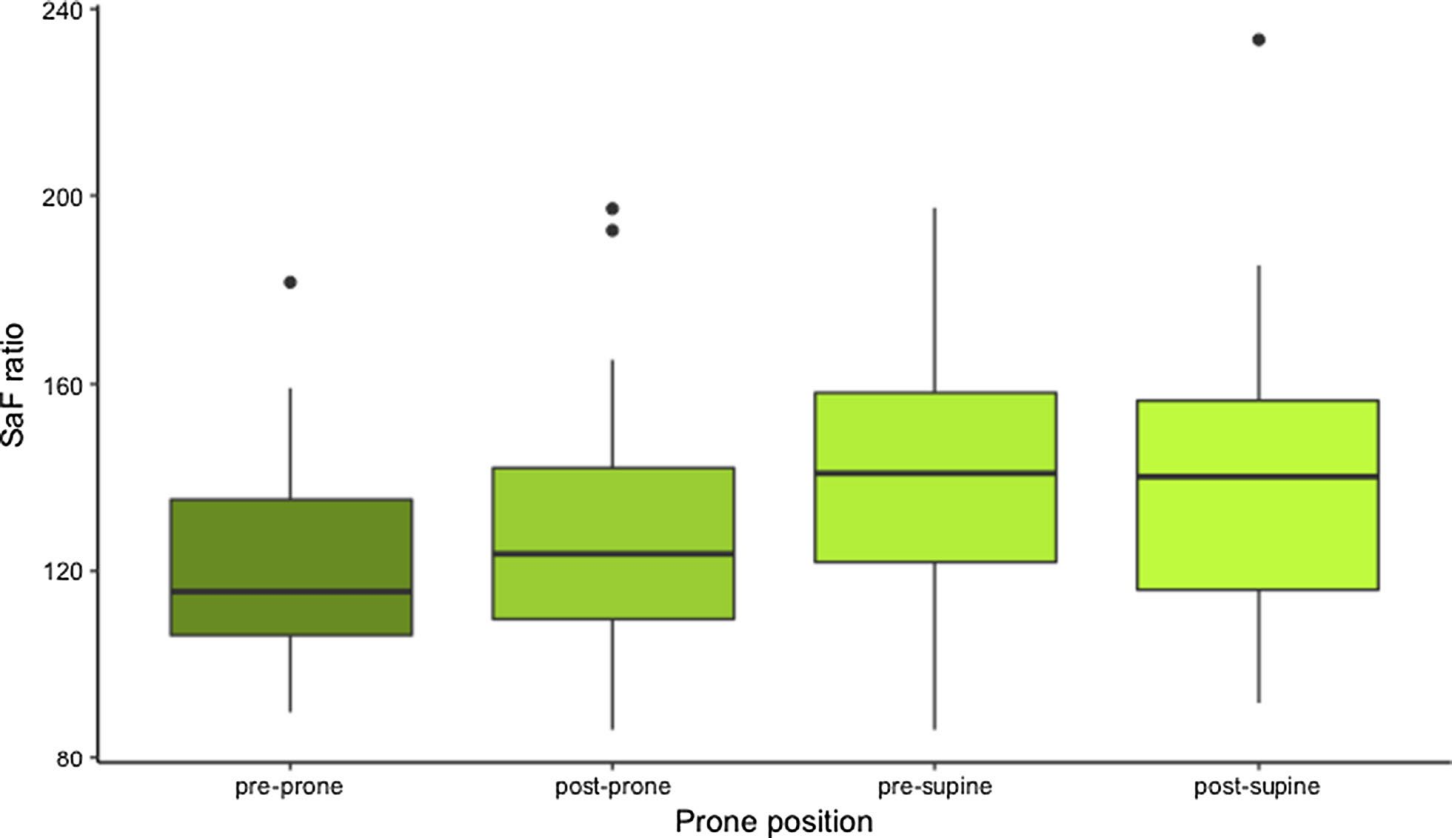
SaF ratio of COVID-19 patients treated with prone positioning (n = 29)

Figures 1, 2, 3 and Additional file 1: Appendix A display the respiratory characteristics (PF ratio, PaCO_2_ and SaF ratio) of patients at each proning stage (pre-prone, post-prone, pre-supine, and post-supine). Oxygenation (PFr) increased with prone positioning and a more modest increase of the PFr persisted after return to the supine position. In addition, there was an increase in the PaCO_2_ throughout the time in the prone position but a return to baseline in the supine position following prone therapy. The SaF ratio demonstrates a similar pattern with oxygenation improving throughout the time in the prone position and an enduring effect when returned supine. Statistical significance for changes in respiratory characteristics across proning are evaluated at a threshold *p* < 0.017, which accounts for multiple testing.

The mean PFr prior to the initial prone positioning was 107.5 ± 5.6 and the first PFr in the prone position, measured 1 h after positioning was 155.7 ± 11.2 (t = 4.62, *p* < 0.001 compared to pre-prone). The PFr after 15 h prone was 142.0 ± 10.8 (t = 3.31, *p* < 0.001 compared to pre-prone) and the PFr after the patients were placed supine was 131.5 ± 9.1 (t = 2.30, *p* = 0.02 (ns) compared to pre-prone). The overall test for difference in PFr across the four proning stages was statistically significant (F = 7.61, *p* < 0.001).

The pre-prone PaCO_2_ was 60.0 ± 2.5 and the 1-h post-prone PaCO_2_ was 67.2 ± 3.1 (t = 0.33, *p* = 0.02 (ns) compared to pre-prone). The PaCO_2_ after 15 h prone was 66.3.0 (t = 2.05, *p* = 0.04 (ns) compared to pre-prone) and the first supine PaCO_2_ measured one hour after prone positioning was 60.4 ± 3.4 (t = 0.13, *p* = 0.90 (ns) compared to pre-prone). The overall test for difference in PaCO_2_ across the four proning stages was not statistically significant after accounting for multiple testing (F = 3.03, *p* = 0.03).

The SaFr prior to the initial prone positioning was 121.3 ± 4.2 and the first SaFr in the prone position, measured 1 h after positioning was 131.5 ± 5.1 (t = 2.28, *p* = 0.03 (ns) compared to pre-prone). The SaFr after 15 h prone was 139.9 ± 5.1 (t = 4.16, *p* < 0.001 compared to pre-prone) and the SaFr after the patients were placed supine was 139.7 ± 5.9 (t = 4.10, *p* < 0.001 compared to pre-prone). The overall test for difference in SaFr across the four proning stages was statistically significant (F = 7.68, *p* < 0.001).

The Pearson’s correlation of SaF ratio with the PF ratio at baseline was r = 0.37. A simple linear regression was fit to predict SaF ratio based on PF ratio. The model R^2^ was 0.108 (F = 4.38, *p* = 0.05) with a β_0_ intercept of 91.0 (*p* < 0.001) and a β_1_ slope of 0.28 (*p* = 0.046). Using these regression coefficients, the SaF ratios predicted by PF ratios of 150 and 200 are 133.2 and 147.3, respectively.

Complications included pressure ulcers in six patients; four on the anterior torso, one related to a mal-positioned nasogastric tube on the upper lip and two with wounds to the cheeks from the tube holder. In these two patients the tube holder was simply inverted, seated on the lower lip and the wounds were treated in a standard fashion. All were stage I or II wounds. There were two episodes of tongue edema, one of which involved hemorrhage. Both were managed by reduction of the edematous tongue to the oropharynx and resolved in the supine position. Neither compromised further positional care. There have been no inadvertent extubations or disruptions of arterial lines, central venous catheters, chest tubes or dialysis catheters.

There were seven unscheduled returns to the supine position. These were due to a mucous plug in the endotracheal tube, a cardiac arrythmia, CO_2_ retention to 138 mmHg and acute hypotension. Only two were evaluated as necessary in a short loop quality review. One unplanned return to supine was due to sudden cardiac arrest not due to the positional therapy or tension pneumothorax.

During the rapid debriefing and performance improvement components of the program we identified more than 30 issues for short loop correction. These opportunities were categorized and immediately addressed in the general areas of communications, procedure, patient selection, information management, equipment, and modifications to the checklist.

## Discussion

We report the process of implementing a safe, effective way to prone hypoxic patients with minimal materials and without disrupting a system already overwhelmed in a disaster scenario. This is not intended to be an outcome assessment or a description of pulmonary pathophysiology but rather a process evaluation of the development and implementation of a quality based protocol for positional care developed and implemented in a stress induced resource limited setting.

The value of prone positioning in severe ARDS has been supported with class I data since the post hoc analysis of the Prone-Supine group in 2001 [1] but was not widely accepted until the PROSEVA trial reported in 2013 [2]. We have had experience with positional therapy for ARDS over many years and in many clinical scenarios [9] but at the time of the COVID-19 surge in New York City there was no experience with routine positional care for ARDS in the NYCHHC/Elmhurst Hospital.

NYCHHC/Elmhurst Hospital Center has been described as the “epicenter of the epicenter” [10] at the time of the peak in deaths in New York State. In addition, Elmhurst is a safety net hospital for the multi-cultural, low-income population of Queens and was further burdened by regional disparities in hospital resources [11]. At that time, the number of ventilated adult patients in the institution had increased from approximately 30 per day to a maximum of 167 in three weeks’ time. In addition, many staff members became ill, newly improvised critical care units were opened and staff were tasked with an increased level of disease acuity and responsibility, all of which severely stressed the medical system. There were unprecedented shortages of RNs, critical care physicians, RTs, critical care units and ICU beds, laboratory capacity, imaging capacity, medications, CPAP equipment, ventilators, vascular access and dialysis catheters and virtually everything else required for the care of patients with severe lung failure.

Our goal was to provide prone positional therapy in an institution that had never operationalized that aspect of pulmonary critical care before. To do so, especially in a time of volume crisis and situational resource limitation, we sought to proceed with several core principles:

1. The positional therapy must be both safe and demonstrably effective.
2. The process must not require any additional work of the physicians, RNs and RTs involved in the care of the patients.
3. Only readily available materials would be used.
4. Our inclusion criteria and clinical processes would reflect the methods of the Class I data producing PROSEVA trial.
5. The program would adhere to principles of HROs.
6. Rigorous adherence to check lists improved continually and in real time and CRM principles would guide all activities from training to practice to evaluations.
7. Intense attention to error in a short loop quality cycle would inform and guide rapid changes and this would be a norm of the team.
8. Concurrent data collection including opportunities for improvement identified in routine post procedure debriefing would inform quality decisions and facilitate protocol evolution.
9. These protocols would be easily adopted by the bedside nurses (RNs) and respiratory therapists (RTs) when the number of critically ill patients returned to a more manageable volume.
10. These methods would be easily adopted in other facilities facing resource limitations, either situationally or in the chronically austere settings common in LMICs.

Basic principles of HROs and CRM guided the evolution of the program and the culture. These principles enabled the team to implement the primary goals with the speed and safety required under the conditions at NYCHHC/Elmhurst during early April 2020. We purposefully designed and led the evolution of the program with a focus on the five traits of HROs: sensitivity to operations, reluctance to oversimplify the reasons for problems, preoccupation with failure, deference to expertise and resilience.

Specifically, these principles informed our decisions as follows. Sensitivity to operations was evident in the realization that we were functioning in an institution with a high volume of COVID patients with severe ARDS and overwhelmed bedside RNs, RTs, and physicians, many of whom did not have training or experience in critical care. The resource limitations in many areas at that time approached those of an austere environment. Reluctance to accept “simple” explanations for problems was a primary motivation for rapid time and point of service debriefings, many short loop correction and re-evaluation cycles and a focus of root causes in the systems domain of error. A preoccupation with failure was best exemplified with a team ethos of an a priori stated concern with “what could possibly go wrong” and a purposefully maintained non-punitive flat hierarchy to facilitate criticisms. We also normalized the use of multiple checklists and the simple use of the word “STOP” at any point by any team member. We deferred to expertise whenever possible recognizing that one program leader brought local cultural, academic, clinical pulmonary/medical perspectives, political strength and legitimacy and longevity to the process and the other provided experiential strength with IPP, clinical surgical/ECMO and severe ARDS perspectives, program development and performance improvement insights and small team leadership skills. The meticulous attention to detail, discipline, and protocol/de-briefing familiarity of the deployed military team members was an additional asset. Finally, this program was developed and evolved in a dynamic and dangerous environment. Many compromises and great flexibility defined both the scenario and the cultural norms of the team. Resiliency is a defining principle of the team. Finally, change was expected and encouraged in rapid sequential quality loops.

The prone team is a cohesive and independent operational team, traits that made it possible to develop rapidly utilizing the principles of CRM. These principles provide a framework for the type of high reliability activity that prone and supine positioning of severely ill and vulnerable patients in a hostile environment characterized by many systems deviations and significant infectious risk to the providers demands. The core principles of CRM that were applicable to this project include a flattened hierarchy, individual and team situational awareness, focus on systems and human errors, non-punitive and immediate feedback, structured communication, crosscheck techniques, and maintaining team integrity and safety.

Our data collection was designed only to show that in this setting IPP improves oxygenation as it does with other types of ARDS and to assure quality of the intervention. Specifically, quality was determined to reflect on the feasibility of a coordinated IPP program during a mass casualty scenario, the rapid operational changes in our procedures and team and patient safety as demonstrated by a low incidence of known minor complications.

In addition to situationally stressed otherwise capable centers this protocol is applicable in settings where resources are traditionally austere. In LMICs these principles may be adapted to local conditions including where ABGs may not be available. Ultimately, all that is necessary is padding of any kind, a pulse oximeter and six strong individuals who can utilize the CRM and HRO team-defining traits to develop and follow a locally appropriate protocol for positional therapy.

This brief report has several limitations. First, our goal was to produce a process report as quickly as was responsible and valid to assist other medical centers in providing positional therapy for the first time despite situationally limited resources. This project was neither designed nor intended to produce outcome data. Many of the patients were still extremely ill and receiving ongoing care, including prone positioning, at the time we chose to report the series. Second, because we only intended to validate the safety and effectiveness of the process, and to produce a rapid report, we chose to report a case series that was small. Third, our measure of the saturation to inspired oxygen (SaF ratio) utilized the measured SaO_2_ instead of the observed peripheral pulse oximeter saturation (SpO_2_). It is a surrogate for an index of oxygenation using only the pulse oximeter (SpF ratio) applicable to more austere settings but, again due to the sheer volume of patients, we were unable to collect those readings with validity. Fourth, our correlation of SaFr with PFr and model fit were only moderately strong and should therefore be interpreted with caution. Moreover, prediction of SaFr based on PFr may not be generalizable to other populations except in concept. Fifth, because we were introducing a seemingly dramatic intervention in an institution that was not familiar with it and that was experiencing multiple extreme stressors, we chose a posture of relative risk aversion regarding patient selection. The one risk we did accept was to position these patients prone with their Hollister ETT holders in place and the ETT in the midline. Under normal conditions this is not advised but we felt that it was necessary. It is preferable to tape the tube and reposition it as indicated and to perform early tracheostomy. At the height of the surge, due to multiple shortages and a temporary moratorium on tracheostomy, which is an aerosol generating procedure, positioning with the tube holder was the only option. Finally, our selection was limited by our belief that even a focused prone positioning team cannot safely manage more than 15 patients with their 30 positional changes and 45 head turns a day so some patients were excluded simply because we did not have time and personnel to safely provide them with IPP.

Patient safety is a critical endpoint of any medical process and the principles of HROs and CRM are frameworks to assure the performance of teams in complex and dangerous environments. This was an environment of chaos and hazard characterized by overwhelmed systems, stressed and variously experienced personnel and an infectious agent that is transmitted by aerosol and respiratory droplets from asymptomatic carriers, has no treatment or vaccine and that has a significant infectivity and mortality. Team safety assumes greater dimensions in a truly hazardous environment. Teams, as well as systems, can suffer trauma just like an individual. Effectiveness and excellence in such a time depends on a culture with an unusual focus on safety with a holistic scope. Initiating a program of positional care in the COVID intensive care units (ICUs) at NYCHHC/Elmhurst required meticulous attention to these principles. Each new situation will require modifications for local conditions, but the protocols and principles described here are transferable to other institutions facing similar challenges.

Although COVID-19 causes a type of severe hypoxemic ARDS that responds to positional therapy like other types of lung failure it remains to be seen if patient important outcomes like mortality can be affected. Those conclusions will require a study design and outcomes data not possible with this report’s intentions and time frame. The data reported simply reflect that positional therapy in this scenario increases oxygenation, only modestly and transiently compromises ventilation, can be conducted with readily available materials, including only pulse oximetry, and an acceptable incidence of known complications.

## Conclusions

We present a program for prone positioning of adult patients with severe hypoxic ARDS due to COVID-19 that can be designed and implemented within days, with a small incidence of known minor complications and demonstrably improved oxygenation during an overwhelming mass casualty scenario. This report describes one simple method to prone hypoxic COVID patients that does not require any additional materials or labor from the already overburdened staff at the bedside and describes the principles of team dynamics that make that possible. This approach may be equally applicable in both traditionally austere environments in LMICs and in otherwise capable centers facing situational resource challenges.

## Supplementary information

**Additional file 1**. **Appendix A**: Respiratory characteristics of COVID-19 patients treated with prone positioning. **Appendix B**: Protocol for Positional Therapy in COVID-19 in a Resource Limited Setting. **Appendix C**: Pre-prone Checklist and Quality Assessment tool. **Appendix D**: Post-prone Checklist.

## Data Availability

The datasets used and analyzed during the current study are available from the corresponding author on reasonable request.

## Abbreviations

ARDS: Acute Respiratory Distress Syndrome
IPP: Intermittent Prone Positioning
COVID-19: Corona Virus Disease 2019
SARS-CoV-2: Severe Acute Respiratory Syndrome Coronavirus 2
HRO: High Reliability Organization
CRM: Crew Resource Management
ABG: Arterial blood gas
PFr: PaO_2_ to FiO_2_ ratio
RN: Registered nurse
RT: Respiratory therapist
SaFr: SaO_2_:FiO_2_ ratio
n: Number % or mean ±
%: Percent
SEM: Standard error of the mean
BMI: Body mass index
CRNA: Certified Registered Nurse Anesthetist
PEEP: Positive end expiratory pressure
PROSEVA: Proning Severe ARDS Patients
ETT: Endotracheal tube
ICU: Intensive care unit
LMIC: Low- and middle-income country

## References

[1] Gattinoni L, Tognoni G, Pesenti A (2001). Effect of prone positioning on the survival of patients with acute respiratory failure. N Engl J Med..

[2] Guérin C, Reignier J, Richard JC (2013). Prone positioning in severe acute respiratory distress syndrome. N Engl J Med..

[3] Gattinoni L, Chiumello D, Caironi P, Busana M, Romitti F, Brazzi L, Camporota L (2020). COVID-19 pneumonia: different respiratory treatments for different phenotypes?. Intensive Care Med.

[4] Robba C, Battaglini D, Ball L, Patroniti N, Loconte M, Brunetti I, Vena A, Giacobbe DR, Bassetti M, Rocco PRM, Pelosi P (2020). Distinct phenotypes require distinct respiratory management strategies in severe COVID-19. Respir Physiol Neurobiol.

[5] Xuefeng Zang, Qian Wang, Hua Zhou, et al. and COVID-19 Early Prone Position Study Group: Efficacy of early prone position for COVID-19 patients with severe hypoxia: a single-center prospective cohort study. *Intensive Care Med.* 2020; 46(10): 1927–29. doi: 10.1007/s00134-020-06182-4 10.1007/s00134-020-06182-4 PMC7375455 32699915

[6] Hines S, Luna K, Lofthus J, Marquardt M, Stelmokas D. *Becoming a high reliability organization: operational advice for hospital leaders*. AHRQ Publication No. 08-0022. Rockville, MD: Agency for Healthcare Research and Quality; 2008.

[7] Haerkens MH, Jenkins DH, van der Hoeven JG (2012). Crew resource management in the ICU: the need for culture change. Ann Intensive Care.

[8] BMJ Quality & Safety - Policy on ethics review for quality improvement reports [online] British Medical Journal, Available at: https://qualitysafety.bmj.com/pages/wp-content/uploads/sites/44/2018/08/Policy-on-Ethic-Reviews2018.pdf. Accessed 25 May 2020

[9] Michaels AJ, Wanek SM, Dreifuss BA, et al.: A protocolized approach to pulmonary failure and the role of intermittent prone positioning. *J Trauma*. 2002;52(6):1037–47; discussion 1047 10.1097/00005373-200206000-00004 12045628

[10] Correal A, Jacobs A, (2020), ‘A Tragedy Is Unfolding’: Inside New York’s Virus Epicenter [Online]. *The New York Times*, Available at: https://www.nytimes.com/2020/04/09/nyregion/coronavirus-queens-corona-jackson-heights-elmhurst.html. Accessed 26 April 2020.

[11] Wadhera RK, Wadhera P, Gaba P (2020). Variation in COVID-19 Hospitalizations and Deaths Across New York City Boroughs. JAMA.

