# Supplementary material for: Prone during pandemic: development and implementation of a quality-based protocol for proning severe COVID-19 hypoxic lung failure patients in situationally or historically low resource hospitals
Source: BMC Pulm Med. 2021 Jan 12;21:25. doi: 10.1186/s12890-021-01401-0 (PMC7802981; doi:10.1186/s12890-021-01401-0)
Supplement: Supplementary file 1 — Additional file 1. Appendix A: Respiratory characteristics of COVID-19 patients treated with prone positioning. Appendix B: Protocol for Positional Therapy in COVID-19 in a Resource Limited Setting. Appendix C: Pre-prone Checklist and Quality Assessment tool. Appendix D: Post-prone Checklist. [file 12890_2021_1401_MOESM1_ESM.docx]

**Appendix A. Respiratory characteristics of COVID-19 patients treated with prone positioning (n = 29)**

|  | pre-prone | post-prone | pre-supine | post-supine |
| --- | --- | --- | --- | --- |
| PF ratio |  |  |  |  |
| Mean (SEM) | 107.5 (5.6) | 155.7 (11.2) | 142.0 (10.8) | 131.5 (9.1) |
| Range | 64.0, 179 | 72.0, 295 | 72.0, 290 | 66.7, 233 |
| t-statistic (p-value) ^a^ | NA | 4.62 (0.000*) | 3.31 (0.001*) | 2.30 (0.02) |
| ANOVA F-statistic (p-value) ^b^ | 7.6144 (0.000*) | | | |
| PaCO2 (mmHg) |  |  |  |  |
| Mean (SEM) | 60.0 (2.5) | 67.2 (3.1) | 66.3 (3.0) | 60.4 (3.4) |
| Range | 39.5, 87.0 | 41.3, 103 | 37.0, 100 | 33.1, 124 |
| t-statistic (p-value) | NA | 2.33 (0.02) | 2.05 (0.04) | 0.13 (0.90) |
| p-value | NA |  |  |  |
| ANOVA F-statistic (p-value) | 3.0321 (0.03) | | | |
| SaF ratio |  |  |  |  |
| Mean (SEM) | 121.3 (4.2) | 131.5 (5.1) | 139.9 (5.1) | 139.7 (5.9) |
| Range | 90.0, 182 | 86.3, 197 | 86.3, 197 | 92.0, 234 |
| t-statistic (p-value) | NA | 2.28 (0.025) | 4.16 (0.000*) | 4.10 (0.000*) |
| p-value | NA |  |  |  |
| ANOVA F-statistic (p-value) ^b^ | 7.6841 (0.000*) | | | |

**Notes:**

Pre-prone = 1 hr before proning; Post-prone = 1 hr after proning; Pre-supine = 15 hr after proning; Post-supine = 1 hr after return to supine position.

^a^ t-statistic is derived from a simple linear mixed model and tests for difference in mean of each characteristic compared to baseline (pre-prone).

^b^ F-statistic is from repeated measures ANOVA and tests the null hypothesis that the four measurements are all equal (e.g., PFr H_0_: PFr _pre-prone_ = PFr _post-prone_ = PFr _pre-supine_ = PFr _post-supine_).

* Bonferroni corrected: p<0.017.

**APPENDIX B**

**Protocol for Positional Therapy in COVID-19 in a Resource Limited Setting**

**Patient Selection**

Inclusion criteria:

Age between 18 and 75 years of age

PaO_2_ to FiO_2_ ratio (PFr) ≤ 150 with:

≤ 72 hours of PFr ≤ 150

Total days of ventilation ≤ 14

Peep of ≥ 5 cm H_2_O and FiO_2_ ≥ 0.60

Exclusion criteria:

BMI > 35

Family or patient decision for no escalation of care

(DNR patients are eligible if clinically appropriate)

Unmanaged abdominal compartment syndrome

CXR with total opacification not due to effusion verified with ultrasound

New (≤ 48 hours) pacemaker insertion

**Materials**

Four standard bed sheets

Three pillows, folded blankets, or folded sheets

8 - 12” for chest

It is essential that this pad is placed only between sternal notch and xyphoid

3 - 6” for pelvis

2 - 4” for face

3 - 4” under ankles

Two large absorbent pads

Tape and ointment for eyes

Pads for exposed boney prominences

Pulse oximeter

New set of EKG leads if appropriate

Intubation equipment available

**Bedside Preparation**

Monitor and EKG leads

Place pulse oximeter on modulated audible setting

Remove all anterior EKG leads and pads

Ventilator

Document current settings with attention to minute volume and pressures

Pre-oxygenate with FiO_2_ of 100%

Airway

Inspect ETT depth at teeth and stabilization

Suction ETT to assure patency and clear any secretions

Skin care

Assure ETT is secure and not threatening skin, lips, or eyes

Carefully pad all boney prominences on body and face

Assure nasogastric (NG) tube does not threaten nasal alae or lip

Inspect tongue for edema or injury potential

Lines and tubes

Discontinue all non-essential lines if possible

Move all lines to midline (or tight to flank) of rotation (to head or feet)

Assure that lines will not end up under the patient

Pad and protect femoral and subclavian lines

Assure that chest tubes are padded, and connections are secure

Communication

Primary care team and bedside RN are informed of plan and timing

Team leader conducts the time out check list

Airway person calls the commands for movement

No other conversations or activities during time out or movement

**Movement Sequence**

The pulse oximeter always remains connected and on audible

Assure the ventral surface of the patient is clear of devices or foreign bodies

Assure all padding is in place

Absorbent pads (sternal notch to knees, top over bottom)

Sheet with cephalad edge rolled towards patient down to the shoulders

This facilitates coverage of the bed once the patient is prone

Two lifting sheets folded in half and lengthwise to 12” width placed perpendicular

One across nipple lines

One across iliac crests

Pillows or folded linen

8 - 12” for chest

3 – 6” for pelvis

adjust to individual habitus to allow belly to hang free of pressure

Second sheet with cephalad edge rolled towards patient to the shoulders

This will become the new bottom sheet

Roll the lateral edges of all linen towards patient until tight

At least two team members on each side

Airway manager counts, maintains airway, never lifts except to stabilize the head

Always roll towards the ventilator

Move efficiently and assume the patient will become volatile but improve when prone

Avoid a lengthy pause in the lateral position

The adjustment of the chest pillows is an immediate priority

**Specific roles and actions**

Airway person

assures all personnel ready

reinforces direction and sequence of movements

demonstrates cadence (i.e., move on three - 1, 2, 3…)

2 persons (or 3 persons) on each side

Move the patient in the sheet package horizontally away from ventilator

Roll the patient back towards ventilator

Center the patient on bed

Clear sheets and absorbent pads from back

Lift chest sling and adjust chest pillows/pads

Center between the sternal notch and the xyphoid

Mild curvature of back, symmetrical, and stable, arms are neutral

Assure belly hangs free

Lift pelvic sling and adjust pads

Flat and symmetrical

Assure belly hangs free

Gentleman’s check to assure genitals are appropriately positioned and padded

Place pad (4” – 8”) under face

Pass suction catheter to assure airway patency of ETT

Inspect for tongue security

Assure ears are flat

Assure the ETT is positioned without compromise

Reattach lines and EKG pads and leads

Reposition foley and chest tubes as necessary

Assure bedside is secure and to the satisfaction of the primary RN

Checklists are read before and after each movement

Immediate debriefing is held, and opportunities identified are recorded

**APPENDIX C**

**Pre-prone check list**

**
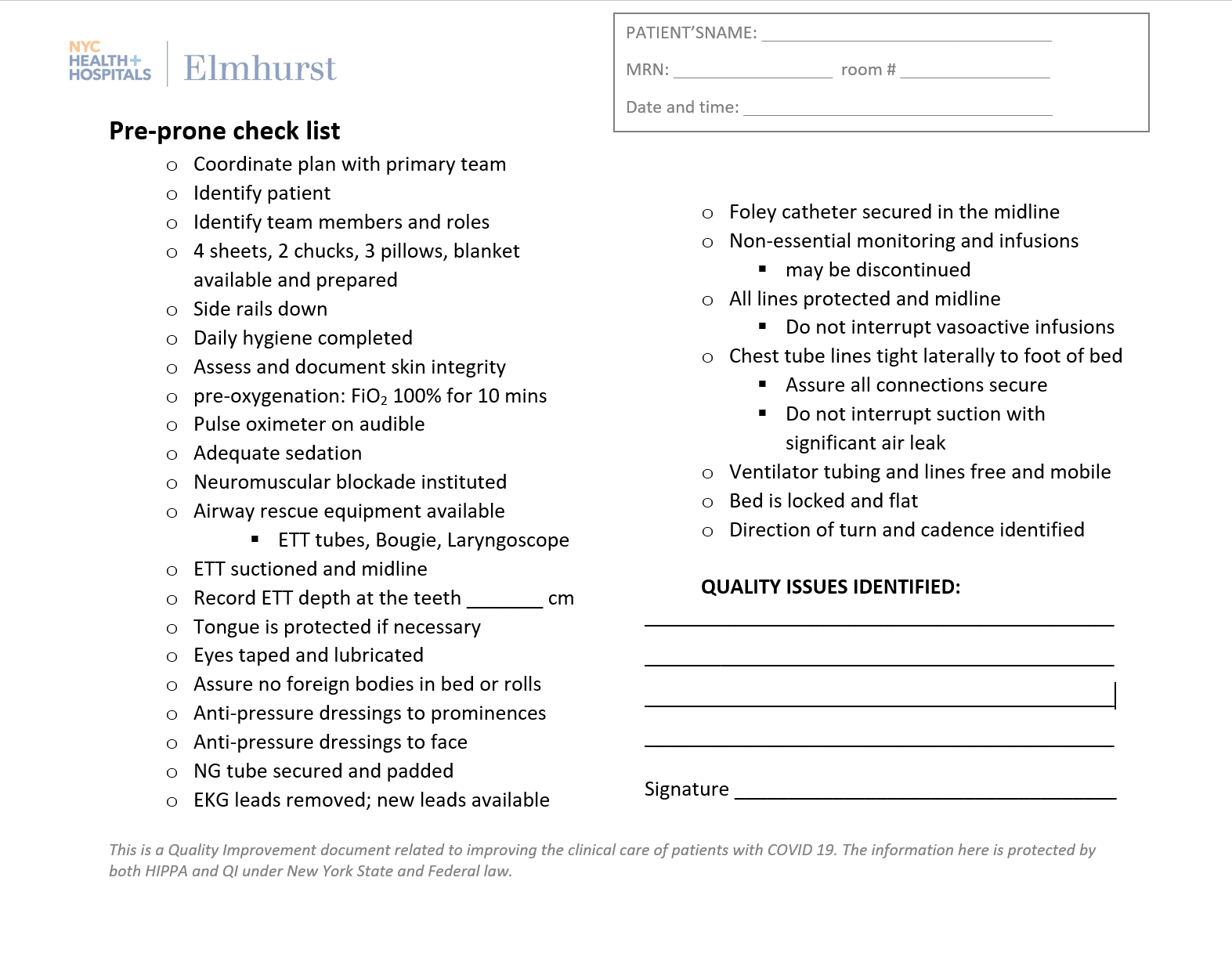
**

**APPENDIX D**

**Post – prone check list**

**
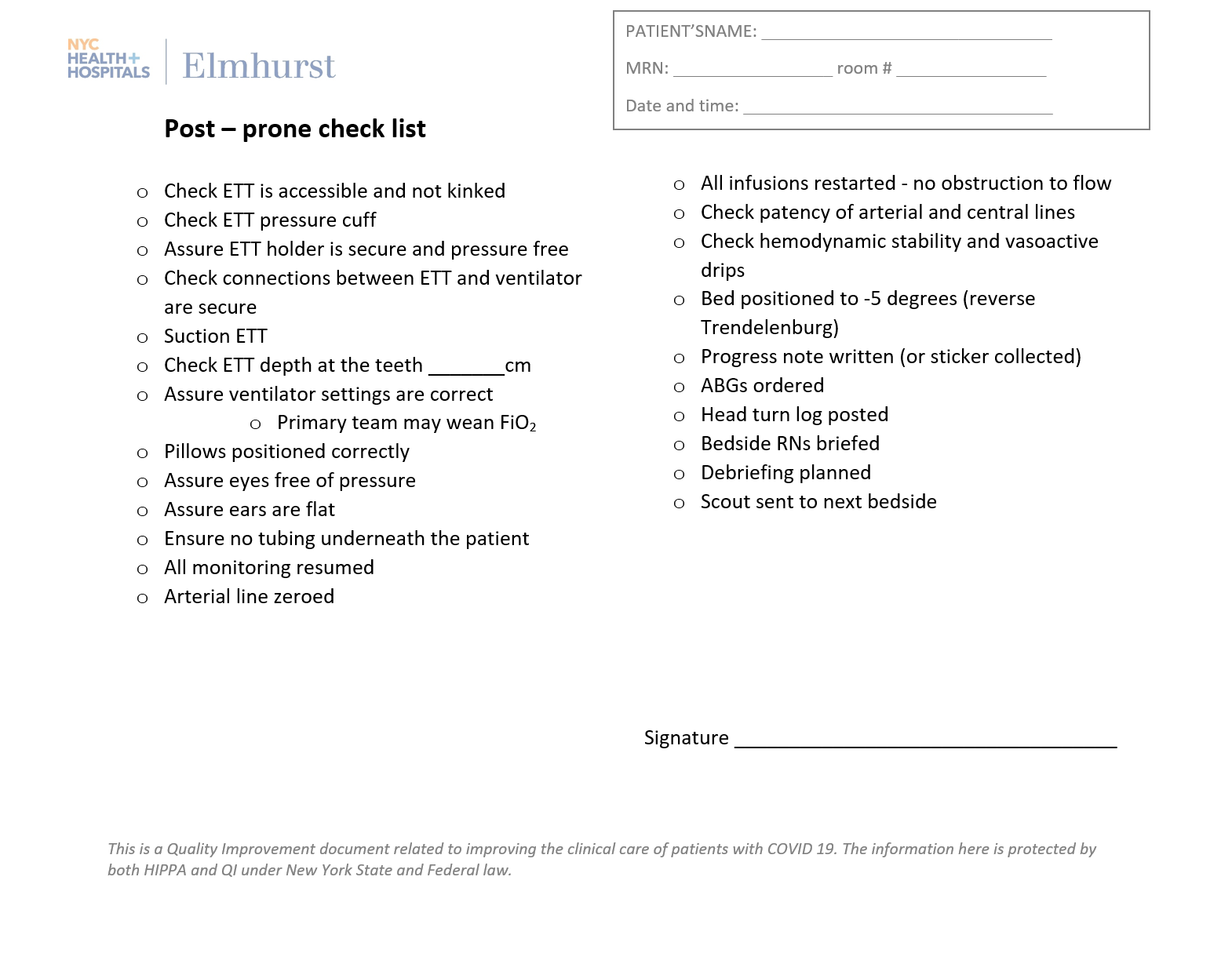
**
